# Lys-ing the Resistance: Targeting Lysosomes to Overcome Chemoresistance in Ovarian Cancer

**DOI:** 10.1007/s11912-026-01742-1

**Published:** 2026-02-05

**Authors:** Aditya Vayalapalli, Balint Kacsoh, Ilana Chefetz

**Affiliations:** 1 College of Liberal Arts and Sciences, Mercer University, Macon, GA 31207 USA; 2 Department of Biomedical Sciences, Mercer University School of Medicine, Macon, GA 31207 USA

**Keywords:** Chemoresistance, Lysosome, Cancer, Sequestration, Autophagy, Exocytosis, Cell death

## Abstract

**Purpose of Review:**

For decades, ovarian cancer (OC) therapy has mainly relied on a regimen of tumor resection followed by treatment with cisplatin and paclitaxel. While this treatment is usually effective initially, resistance to this regimen in OC is widespread and is often the cause of death in OC patients. In the attempt to find new molecular targets for the treatment of chemoresistant OC, understanding the precise mechanisms of chemoresistance remains a paramount task. This review examines the critical roles of the lysosome in the instigation of chemoresistance in OC and explores possible clinical applications for overcoming chemoresistance.

**Recent Findings:**

Lysosomes contribute to chemoresistance through various mechanisms, including increased lysosomal biogenesis, resulting from the enhanced activity of transcription factor EB, a master regulator of the autophagy-lysosome pathway, which enhances cellular capacity for drug sequestration. Lysosomal exocytosis allows the cell to secrete chemotherapeutic agents from OC cells. Lysosomal autophagy pathways enable OC cells to selectively recycle cell components during chemotherapeutic stress. Finally, lysosomal signaling pathways disrupt various cell death mechanisms such as apoptosis, necroptosis, and ferroptosis, which allow cancer cells to evade death under chemotherapeutic stress.

**Summary:**

Targeting lysosomal biogenesis, stage-specific autophagy modulation, and lysosome-dependent metabolic vulnerabilities are promising avenues for sensitization of chemoresistant OC cells.

## Introduction

Despite accounting for only 4% of cancers in women, ovarian cancer (OC) is the deadliest gynecological malignancy [1, 2]. OC treatment has relied on a classic regimen of tumor resection followed by treatment with cisplatin and paclitaxel for several decades [2, 3]. While most patients initially respond to this treatment, chemoresistance emerges in around 70% of patients, which is characterized by the return of the cancer within six months of the completion of the initial chemotherapy regimen [1]. Resistance can also be primary, where the cancer does not respond to the initial chemotherapy [3]. Chemoresistant OC is usually treated with the second-line therapeutic doxorubicin, which causes cardiotoxicity and has low response rates. Due to the multifaceted difficulties of treating chemoresistant OC and the lack of effective treatments for this disease, the five-year survival rate for OC patients diagnosed with chemoresistance is around 40% [1, 4]. The development of the chemoresistance phenotype is a multifactorial process, often correlating with biological factors that fall into three main categories: processes that (1) prevent the chemotherapeutic from reaching its target in the cancer cell, (2) metabolize the chemotherapeutic to render it inert, or (3) reduce the ability of the cancer cell to go through cell death processes once resistance has been established [3–5]. Among the various molecular mechanisms of chemoresistance in OC, lysosomes have emerged as key players that enable OC cells to evade cell death under chemotherapeutic stress.

Lysosomes are membrane-bound organelles that play a major role in the degradation and recycling of unnecessary or dysfunctional cellular components [6–8]. The integration of cellular metabolism and stress response mechanisms in this organelle makes it deeply interconnected with all cellular processes, including autophagy, DNA repair, and cell transport [6–8]. The lysosome maintains an acidic pH of around 4.5, compared to the cytosolic pH of 7.2, and contains various hydrolytic enzymes, including lipases, nucleases, and proteases, which target and break down cellular components [6–8]. The lysosome is also highly adaptive, utilizing information from other cellular structures to control anabolic and catabolic processes. This organelle also regulates nutrient levels within the cell, targeting the rapamycin complex 1 (mTORC1) and adenosine monophosphate-activated kinase (AMPK) pathways [6–8].

Lysosome-mediated chemoresistance, in general terms, is the alteration of lysosomal pathways such as autophagy and lysosomal exocytosis to help cancer cells evade the effects of chemotherapy. Lysosomes may sequester chemotherapeutic agents, effectively keeping them away from their intracellular target. Cancer cells may also exploit the lysosome-autophagy pathway to break down organelles that are not essential for proliferation to help the cell survive and recover from chemotherapeutic stress (Tables 1 and 2).

**Table 1:** Genes influencing lysosome-mediated chemoresistance based on TCGA data

Gene	Function	Contribution to OC chemoresistance
Lysosomal Biogenesis & Transcriptional Control of lysosomal proteins		
Transcription factor EB	activates lysosome formation genes, upregulates lysosomal biogenesis	upregulation/increased nuclear translocation increases lysosomal capacity for drug sequestration
Beta Glucosidase	lysosomal hydrolase, breaks beta-glycosidic bonds	overexpression suppresses AKT/mTOR and enhances nuclear translocation of TFEB
Pyridoxamine 5’-Phosphate Oxidase (PNPO)	activates Vitamin B6	overexpression increases lysosomal number via TFEB, knockdown sensitizes OC to paclitaxel
Lysosome Associated Membrane Protein 1	marker of lysosomal activity	knockout of LAMP1 reverses PNPO-mediated chemoresistance
Lysosomal Sequestration and Exocytosis		
Transient Receptor Mucolipin 1	lysosomal calcium channel, regulates lysosomal exocytosis	upregulated in chemoresistant OC, regulates lysosomal exocytosis
Ras-associated Binding Protein 27 A	regulates vesicular exocytosis	upregulated in cisplatin-resistant OC, regulates lysosomal exocytosis & exosomal secretion of chemotherapy
Ras-associated Binding Protein 7	involved in lysosomal trafficking and lysosomal exocytosis	downregulated in cisplatin-resistant OC, which skews OC cell toward exocytosis
Hypoxia-inducible Factor 1-alpha	promotes cell survival under low O2 levels	mediator of cancer adaptation to hypoxia, increases chemotherapy secretion via exosome
NK Homeobox 3-2	regulates skeletal development and chondrogenesis	high expression correlates with worse OC outcomes, autophagic destruction of this protein may lead to OC sensitization
p53	regulates cell growth and division, prevents cancer	regulates autophagy-lysosome degradation of NKX3-2, often dysregulated/mutated in cancer
Autophagy and Lysosomal Signaling		
Extracellular Signal-regulated kinases	transmits signal from cell surface to nucleus	promotes autophagosome-lysosome fusion through UVRAG-Rab axis, enables pro-survival autophagy under chemotherapeutic stress
BIR Repeat-containing ubiquitin conjugating enzyme	involved in ubiquitination, inhibits apoptosis	depletion enhances chemoresistance through precision autophagy by AMPK-ULK1 axis
Adenosine Monophosphate Kinase	phosphorylates AMP	activates autophagy through mTORC1 inhibition, signaling enhanced by BRUCE depletion
Mechanistic Target of Rapamycin Complex	regulates cell metabolism, growth, survival	suppresses autophagy & TFEB activity, suppression under stress induces autophagy
Argininosuccinate synthase 1	processes extra nitrogen into urea, helps synthesize arginine	decrease in expression/loss of this gene in resistant OC creates extracellular arginine dependence, possibly inhibits chemoresistant OC
Microtubule-associated protein 1 A/1B chain 3B	bind and stabilize microtubules	marker of autophagic flux, high LC3B + low NKX3-2 correlated with better survival
Cell Death Pathways and Lysosomal Signaling		
Cathepsin L	protein degradation, apoptosis, processing of antigens	overexpression associated with chemoresistance, knockout induces apoptosis in chemoresistant OC cells
Cathepsin D	protein degradation, apoptosis, activation of zymogens	vital for apoptotic activation from cisplatin, proapoptotic when used in conjunction with lysosome membrane permeabilization
Cathepsin B	intracellular protein degradation and processing	released during necroptosis, promotes cell death
Receptor Interacting Protein Kinase 1	maintains balance between cell survival and death	no studies specifically connecting this to OC chemoresistance
Mixed Lineage Kinase Domain-like protein	key regulator of necroptosis	upon phosphorylation, translocates to lysosome and causes LMP & release of cathepsin B
Aldehyde Dehydrogenase	detoxifies aldehydes, converts into less toxic carboxylic acids	marker of OC stem cells
Janus Kinase 1	necroptosis-associated gene	upregulation correlates with worse OC prognosis
Brain-type Glycogen Phosphorylase	necroptosis-associated gene	upregulation correlates with worse OC prognosis
Signal Transducer and Activator of Transcription 1	necroptosis-associated gene	upregulation correlates with worse OC prognosis

Twenty-six key genes that drive lysosome-mediated resistance to chemotherapy in ovarian cancer. These molecules could be evaluated for therapeutic potential in clinical intervention

**Table 2:** Lysosome-related prognostic genes

Gene	Function	Upregulated or Downregulated in High Disease- free Survival Group	*P* Value
Overall survival			
CLTA	Delivers material to lysosome for degradation	Upregulated	0.03
PSME1	Activates proteasome	Upregulated	0.08
RABAC1	Mediates lysosomal exocytosis through influencing Rab 11 GTPase	Downregulated	0.004
Disease Free Survival			
SLC38A9	Lysosomal amino acid transporter, activates mTORC1 signaling, arginine sensor	Downregulated	0.0051
RAB33B	Fusion of autophagosome with lysosome	Downregulated	0.0063
TMEM45A	Induction of lysosomal membrane permeabilization	Fluctuated between upregulated and downregulated states	0.019
SORT1	Directs proteins to lysosome for degradation	Upregulated	0.011
VCP	Lysosomal structure and function, lysosomal biogenesis, autophagy	Upregulated	0.0096

This table identifies specific lysosomal genes whose expression in a high disease-free survival group correlates significantly with overall survival or disease-free survival. This table is not a comprehensive list of all prognostic lysosomal genes for OC

The lysosome’s therapeutic potential in the context of restoring chemosensitivity is vastly underexploited, and more research should be done to understand how the lysosome’s metabolic and regulatory abilities might be modified to address chemoresistance to improve disease-free and total survival (Table 3).

**Table 3:** Clinical trials of lysosome-targeting drugs to treat cancer

NCT05634707	Fluoxetine (Prozac)	IDHwt glioma	Recruiting	To assess whether fluoxetine can induce lysosomal stress
NCT04735068	Binimetinib and Hydroxychloroquine*	KRASmut Non-Small Cell Lung Cancer	Completed	To evaluate the use of hydroxychloroquine (HCQ) along with binimetinib
NCT01023737	Vorinostat and Hydroxychloroquine*	Colorectal or renal	Completed	To evaluate the use of hydroxychloroquine (HCQ) along with vorinostat
NCT01587144	Lucanthone (Lysosome membrane disruption)	Glioblastoma Multiforme	Terminated	To determine the effectiveness of lucanthone, with temozolomide
Preclinical	Ifenprodil	Glioblastoma	NA	To induce lysosome leakage and apoptosis
Preclinical	Amoxapine	Glioblastoma	NA	To induce lysosome leakage and apoptosis

*More drug combinations with Hydroxychloroquine are being evaluated in clinical studies

Trials evaluating lysosomal modulation as a therapeutic strategy for chemoresistant cancers through autophagy inhibition, induction of lysosomal stress, and lysosomal membrane disruption

## Lysosomal Biogenesis

Alterations to lysosomal biogenesis and its transcriptional control are a key mechanism through which OC cells can resist chemotherapy. Transcription Factor EB (TFEB) is a cytoplasmic protein that translocates to the nucleus and activates the transcription of genes involved in the upregulation of lysosomal biogenesis [6, 8, 9]. mTOR-mediated phosphorylation of TFEB prevents the transcription factor from entering the nucleus [10]. Downregulation/inhibition of mTOR allows TFEB to be in its unphosphorylated state, allowing nuclear translocation [10].

In chemoresistant OC, increased expression and activity of lysosome-related proteins contribute to chemoresistance through enhancement of drug sequestration systems. In one study, cisplatin-resistant IGROV and A2780 cells exhibited enhanced nuclear translocation of TFEB upon exposure to cisplatin compared to a sensitive control, which indicated chemotherapy-induced cellular stress selectively activates TFEB activity in resistant OC cells [11]. Increased lysosomal biogenesis means greater capacity and efficiency of lysosomal drug sequestration, allowing for the generation of a robust chemoresistance phenotype.

**Fig. 1:**
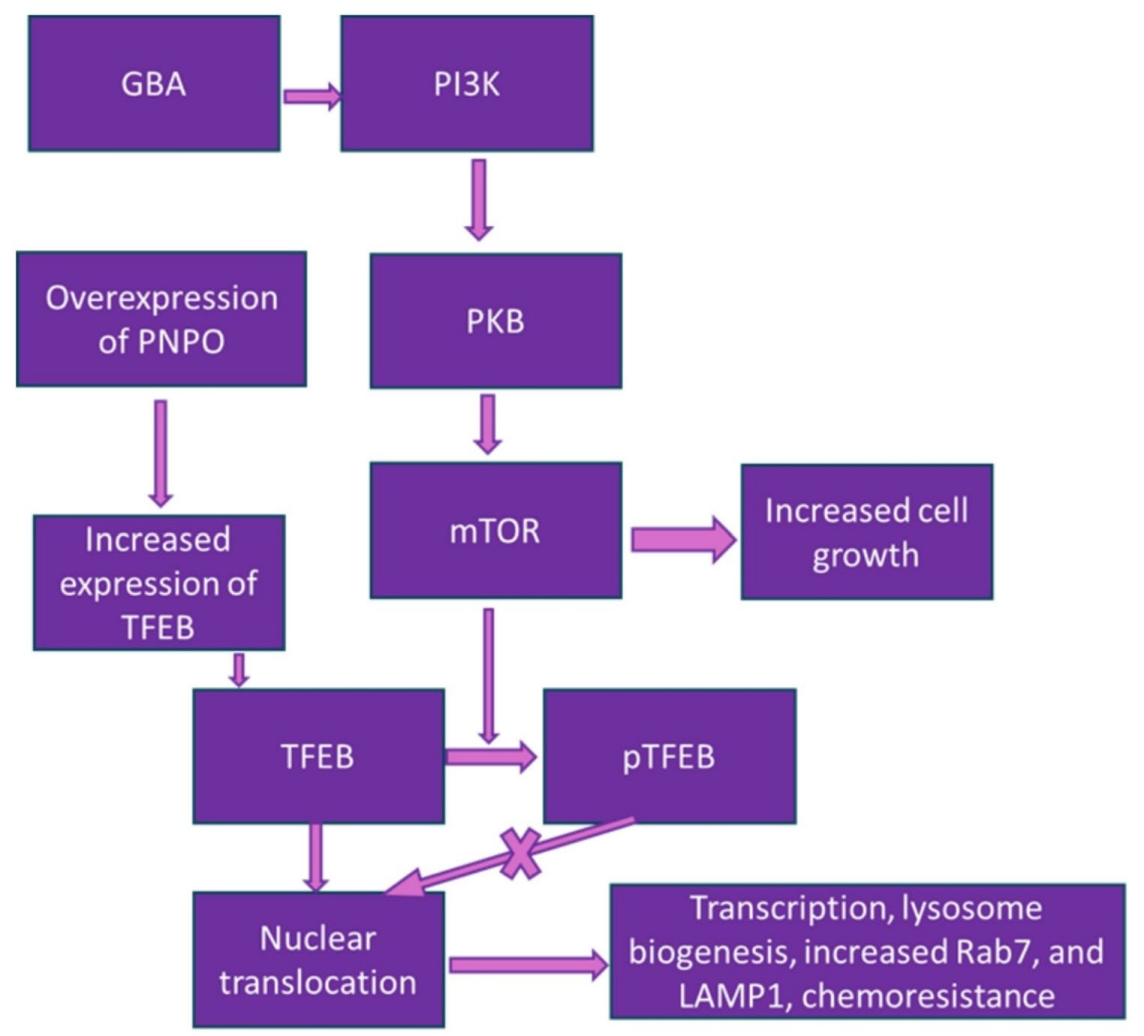
GBA effect on cancer cell proliferation, chemoresistance, and lysosome biogenesis

Beta-glucosidase (GBA) is a lysosomal hydrolase that breaks the beta-glycosidic bonds that hold monosaccharide units together [12]. Its overexpression has been observed in OC, as determined by data from the Cancer Genome Atlas [13]. The same study also assessed SKOV-3 and Caov-3 cell lines treated with cisplatin in vitro, and found that cells with overexpressed GBA had a higher level of survival [13]. When GBA was knocked out, decreased proliferation and higher levels of apoptosis were observed [13]. This effect of decreased GBA was possibly mediated by suppression of the PI3K/Akt/mTOR signaling pathway, as is the case in breast and ovarian cancer [13, 14]. GBA does not directly influence the Akt/mTOR signaling pathway. However, it plays a role in regulating the availability of glucose, which can indirectly affect the pathway by impacting mTOR activity (Fig. 1).

Akt/mTOR (mostly mTOR1) usually phosphorylates and inactivates TFEB (prevents nuclear translocation), so the suppression of this pathway by inhibiting GBA will decrease TFEB inactivation through maintenance of the unphosphorylated state, allowing nuclear translocation [13]. Theoretically, this would upregulate lysosomal biogenesis and activity and, by proxy, chemoresistance in OC cells.

The inhibition of the GBA pathway is not the only means by which TBEF is leveraged to induce drug resistance in chemoresistant OC. Pyridoxine 5’-phosphate oxidase (PNPO) is a regulatory protein that converts vitamin B6 into its active form. Some evidence points to PNPO controlling resistance to paclitaxel [15]. Overexpression of PNPO in OVCAR-3, SKOV-3, and A2780 cell lines increased the number of observed lysosomes and upregulated expression of genes associated with lysosomal enzymes and membrane transporters, which was mediated by increased expression of TFEB [15]. Silencing of PNPO inhibited tumor growth and sensitized tumors to paclitaxel [15]. The role of PNPO-lysosome interaction in the mediation of chemoresistance effect was demonstrated by the reversal of PNPO’s protective effects against paclitaxel when lysosome-associated membrane protein (LAMP1) was suppressed [15]. PNPO overexpression in chemoresistant OC cells originated from the SKOV-3 and OVCAR-3 cell lines, also facilitated autophagic flux through increases in lysosomal function, without affecting the induction of autophagy [15]. This enabled better degradation of damaged cell structures during chemotherapy-induced stress. Notably, this effect appeared to target later stages of autophagy [15].

### Lysosomal Sequestration

**Fig. 2:**
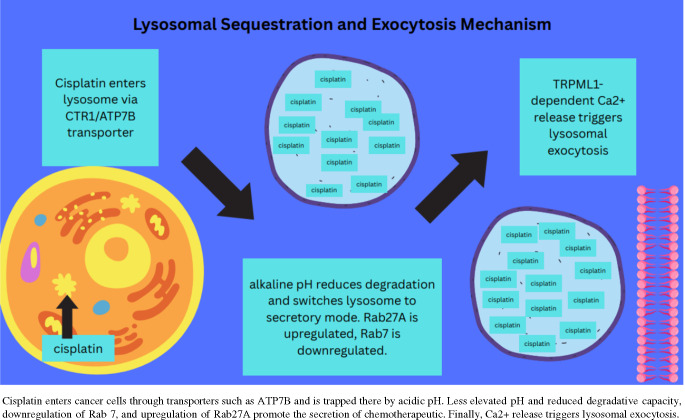
Lysosomal Sequestration and Exocytosis Mechanism

The lysosome’s ability to sequester and excrete chemotherapeutic agents is a key axis by which chemoresistant cancer cells defend themselves against chemotherapy [16, 17]. In OC, this mechanism of chemoresistance is prominent, specifically when cisplatin is actively shuttled into lysosomes by copper transporters (CTRs) such as ATPase copper transporter Beta (ATP7B). Once inside the lysosome, cisplatin may be either degraded or removed by lysosomal exocytosis. In lysosomal exocytosis, lysosomes fuse with the plasma membrane and release any chemotherapeutic agents they captured into the extracellular environment [18]. This mechanism is tightly regulated by transient receptor potential mucolipin 1 (TRPML1) lysosomal calcium channels [18] (Fig. 2). A study that assessed TOV21G and OVCAR8 OC cell lines found that TRPML1 was upregulated in chemoresistant cells following in vitro incubation with cisplatin [18]. In the same study, when TRPML1 was inhibited using ML-SL1, which blocks TRPML1 channel activity, or the gene encoding TRPML1 was silenced, lysosomal exocytosis and lysosomal biogenesis were inhibited, which caused cell death in chemoresistant OC cells [18]. These findings illustrate the complex nature of lysosome-mediated chemoresistance, where sequestration and exocytosis work together to minimize the damage caused by chemotherapeutics to OC cells.

Increased lysosomal pH causes lysosomes to switch from a more degradative function to a secretory one [19]. Some chemoresistant cells also display reduced lysosome number and acidity, which suggests a shift in their function from degradation to export [19]. When the lysosome has more functional degradative pathways, chemotherapeutic agents are captured by multivesicular endosomes, which would send them to the secondary lysosome (or endolysosome, the more acidic late endosome fused with the primary lysosome) for degradation. In the less acidic environment with less degradative and more secretory function, the chemotherapeutic-containing multivesicular endosome fuses with the primary lysosome, which then fuses with the plasma membrane, effectively secreting the chemotherapeutic rather than degrading it [19]. The underlying mechanism involves an imbalance in Ras-associated binding protein (Rab) GTPase activity, which regulates vesicular exocytosis [19]. Rab27A was found to be upregulated in cisplatin-resistant OC cells, likely due to its induction of cell export pathways [20]. Similarly, Rab7 (a marker of the late endosome) was found to be downregulated in cisplatin-resistant OC cells, likely because Rab7’s downregulation also promotes lysosomal exocytosis of cisplatin by skewing lysosomal vesicle fate towards exocytosis and away from degradation [10, 21]. Rab7 may also play a role in cisplatin chemoresistance in OC through its regulation of the endosomal pathway, lysosomal biogenesis, and extracellular vesicle secretion [22].

Higher levels of transcription factor NK homeobox 3 (NKX3-2) are correlated with chemoresistance and increased mortality in OC [23]. NKX3-2 is degraded by the autophagy-lysosome pathway, which is regulated by p53 [23]. Data from the Cancer Genome Atlas showed that patients with a low level of NKX3-2 and high expression of microtubule-associated protein 1 A/1B chain 3B (LC3B), which marks autophagic flux, have better overall survival [23]. This suggests that lysosome-mediated clearance of NKX3-2 may suppress chemoresistance [23]. The degradation of NKX3-2 depends on functional lysosomal trafficking, which is mediated by Rab7 and p53. The study concluded that therapeutic strategies to restore lysosomal function through increased Rab7 or reactivation of p53 could sensitize OC cells to general chemotherapy treatment [23]. In cancer cells, hypoxia triggers a variety of cellular and molecular adaptations that promote cancer cell survival, proliferation, and metastasis [24]. In hypoxic conditions, endolysosomal fusion is diminished, which enhances chemoresistance through increased exosome release [24]. Hypoxia-inducible factor-1alpha (HIF-1α) plays a central role in this adaptation [25, 26]. An extract of the plant *Scutellaria baicalensis* counteracted hypoxia-induced chemoresistance by increasing the lysosomal degradation of HIF-1alpha [25, 26]. This made the OC cells sensitive to chemotherapeutic [25]. When the lysosome activity was inhibited with chloroquine (due to an increase in pH), the sensitization effect of the *Scutellaria baicalensis* diminished, highlighting the effect of the lysosome in reversing chemoresistance in hypoxic OC [25].

## Pro-survival Autophagy and Lysosomal Signaling Under Chemotherapeutic Stress

Autophagy is the lysosome-mediated process by which cells endure stress through the selective recycling of cell components [27]. This process, which is consistently upregulated in cells exposed to chemotherapeutic agents, allows for the maximization of the survival chances of OC cells exposed to chemotherapeutic agents [28, 29].

In OC, high levels of autophagic activity directly correlate with higher earlier-stage chemoresistance, and this effect is diminished as other chemoresistance mechanisms evolve. In these early stages, autophagy serves to buffer stress and give OC cells time to form more sustainable adaptations against chemotherapeutics [29]. Once chemoresistance is established, levels of autophagic stress may decline. This highlights the importance of autophagy’s role in the onset of chemoresistance rather than the maintenance of the phenotype. Clinically, this may mean that treatment with autophagy inhibitors should be pursued as a prophylactic treatment to prevent chemoresistance when treating patients during their first round of chemotherapy.

In this line of reasoning, mechanistic studies have identified specific molecular targets for autophagy inhibition. Inhibition of extracellular signal-regulated kinases 1/2 (ERK1/2) prevented the fusion of the autophagosome and lysosome via the ERK1/2- UV radiation resistance-associated gene (UVRAG)-Rab axis, which in turn stopped autophagic flux and increased cell death rate in OC cells treated with cisplatin and paclitaxel. This finding highlights the singular importance of autophagy suppression, specifically through ERK1/2-targeted strategies, to prevent resistance and improve treatment outcomes. This effect might be model-dependent, as ERK1/2 inhibition in OvCAR8 and PEO4 cell lines caused enrichment of OC stem cells (OCSCc), potentially due to triggering autophagy [30].

OCSCs are incredibly resistant to chemotherapy treatment and contribute heavily to the generation of the OC chemoresistance phenotype [31–34]. OCSCs likely rely on lysosomal function, specifically autophagy, for self-renewal and survival [35]. Disruption of autophagy with synthetic K+/H + exchangers to inhibit autophagy selectively killed OCSCs from HEYA8 and SKOV-3 cell lines without affecting healthy OC tissues, which indicates that targeted inhibition of lysosomal pumps could be a promising means of generating more specific treatments for OC [35].

The BIR repeat-containing ubiquitin conjugating enzyme (BRUCE) is an enzyme that transfers ubiquitin onto proteins, playing a major role in precision autophagy by labeling specific proteins for targeting by the lysosome [35]. In one experiment, the depletion of BRUCE in U2OS OC cells induced chemoresistance through the triggering of AMPK/Unc51-like autophagy kinase 1 (ULK1) signaling, which enhanced lysosomal enzyme activity and the ultimate increase in autophagy (Che et al., 2019). Conversely, when autophagy was inhibited, depletion of BRUCE did not lead to the chemoresistance phenotype [36]. The chemoresistance phenotype was decreased through inhibition of autophagy using chloroquine, indicating that BRUCE-mediated cell survival under chemotherapeutic stress relies on functional lysosomal pathways [36]. In a later experiment, knockdown of BRUCE in PEO4 OC cells improved the ability of the OC cells to resist cisplatin as well as their autophagic ability, indicating autophagy’s contribution to cisplatin resistance in OC cells [36].

Metabolic adaptation is a major way in which OC increases growth, proliferation, and metastatic rate, especially in response to chemotherapeutics [37]. SKOV-3 cells that were cultured in arginine-free medium, which slowed the growth of the cells but did not kill them, exhibited increased autophagy to recycle cell components as sources of arginine [38]. In the same study, OC cells grown in arginine-free medium with inhibited autophagy induced by either the agent chloroquine or through post-transcriptional suppression of Bcl-2 interacting protein 1 (BNIP1) displayed reduced proliferative ability even after exposure to normal medium with arginine [38].

In OC, arginine deprivation induced autophagy through suppression of mTORC1, which promoted the chemoresistance phenotype [38]. Relapsed tumors also tend to stop expression of a key enzyme in the synthesis of arginine, argininosuccinate synthase 1, causing dependence on extracellular arginine and inability to survive in arginine-poor environments [38]. However, this dependence on arginine can only be leveraged when autophagy is suppressed, possibly because chemoresistant OC uses autophagy to degrade cell components to generate free arginine, thus underscoring the centrality of lysosomal processes in OC chemoresistance [38].

Lysosomal enzymes such as the protease cathepsin D (CD), which nonspecifically hydrolyze many different polypeptides, are also repurposed in resistant OC cells to evade apoptosis under chemotherapeutic stress [39]. In chemosensitive cells, lysosomal membrane permeabilization leads to the release of CD into the cytosol, where the enzyme activates the pro-apoptosis protein Bax [40]. Lysosomal membrane permeabilization is crucial in this axis; in chemoresistant cells, when CD levels are high, the lysosomal membrane remains intact, and cells avoid apoptosis. CD can therefore cause or inhibit chemoresistance, depending on the level of lysosomal integrity inside the cell [40].

## Lysosomal Signaling, Cell Death, and Chemoresistance

A lack of cell death response, even when a chemotherapeutic successfully fulfills its role in causing cellular damage, plays a major role in the induction of chemoresistance. The lysosome interplays with apoptosis, ferroptosis, and necroptosis in various ways that lead to the generation of the chemoresistance phenotype.

Apoptosis is the main form of programmed cell death in cancer and is driven in part through the action of cathepsins, lysosomal proteases that are released to break down cellular machinery once apoptosis is activated [41–47]. Certain cathepsins have been shown to play roles in the induction of the chemoresistance phenotype [46, 47]. One study found that overexpression of cathepsin L is associated with chemoresistance, and two studies found that cathepsin L knockdown in SKOV3 and TAX cells increased apoptosis in chemoresistant cell lines [46, 47]. Cathepsin D seems to show the reverse relationship, with one study finding that its overexpression correlated with p-53-dependent apoptosis in OC cells and another study finding that cathepsin D is vital for the activation of apoptosis pathways in OC cells from PA1-neo ovarian teratocarcinoma, A2780, and SKOV3 cell lines exposed to cisplatin [40, 48]. Overall, more research is needed to generate a better understanding of how cathepsins mediate apoptosis in chemoresistant and sensitive OC. Another possible mechanism of apoptosis-related lysosome-mediated chemoresistance in OC lies in lysosomal permeability, as described earlier.

In addition to apoptosis, there is some preliminary evidence tying the alternative cell death pathways, necroptosis and ferroptosis, to lysosome-mediated chemoresistance in OC.

Necroptosis is a form of programmed cell death that occurs via the formation of the necrosome through complexing of receptor-interacting protein kinase 1 (RIPK1) and RIPK3 [49–52]. The necrosome formation causes phosphorylation of mixed lineage kinase domain-like protein (MLKL), which triggers cell death through the instigation of membrane damage [53]. Phosphorylation of MLKL also promotes translocation of the protein to the lysosomal membrane, where it increases the permeability of the lysosomal membrane, leading to the release of cathepsin B, which promotes cell death as well [54, 55]. Disruption to lysosome-mediated autophagic flux, or the process by which autophagosomes are formed, also plays a role in necroptosis and can therefore also become a means by which necroptosis in chemoresistant OC is activated. When autophagic flux is blocked, the accompanying accumulation of nonfunctional autophagosomes generates a scaffold for the assembly of necrosomes [56, 57]. Similarly, when autophagic flux is more efficient, RIPK proteins and necrosome components get degraded at a higher level [58–61]. Therefore, the blockage of autophagic flux provides a promising clinical opportunity to cause cell death in chemoresistant cells through blockage of autophagic flux.

Ferroptosis is another alternative mechanism of cell death that utilizes iron-dependent lipid oxidation to form lipid peroxides that generate cascades of reactive oxygen species that damage the cell membrane [62, 63]. Lysosomes play a major role in the re-mobilization of intracellular iron, which is crucial for the execution of ferroptosis, and lysosomes are currently being considered as therapeutic targets in cancers. Emerging evidence also shows that treatments that affect ferroptosis via iron metabolism may be a critical pathway to exploit in the treatment of chemoresistant gynecological malignancies [64, 65]. The agent erastin, which can induce both necroptosis and ferroptosis, has been used successfully in conjunction with the chemotherapeutic cisplatin to treat OC, but chemoresistance to this drug also poses a threat to the successful eradication of OC [66, 67].

## Conclusion

Through its roles in the mediation of autophagy, drug sequestration and efflux, and targeted degradation, the lysosome is a major player in the instigation of the chemoresistance phenotype in OC. Key mechanisms in lysosome-mediated chemoresistance in OC include TFEB-mediated increases of lysosomal biogenesis that decrease the cytosolic concentration of chemotherapeutic agents, autophagic recycling of cellular components that help deal with chemotherapeutic-induced stress, and lysosomal degradation that allows key metabolic adaptations in chemoresistant OC.

In the clinical setting, lysosomal biogenesis, precise autophagic modulation that depends on the stage of cancer, and exploitation of lysosome-mediated metabolic deficiencies may be useful for the exploitation of selectively sensitizing OC cells.

## Key References

- Cerda-Troncoso, C., Grünenwald, F., Arias-Muñoz, E., Cavieres, V. A., Caceres-Verschae, A., Hernández, S., Gaete-Ramírez, B., Álvarez-Astudillo, F., Acuña, R. A., Ostrowski, M., Burgos, P. V, & Varas-Godoy, M. (2024). Chemo-small extracellular vesicles released in cisplatin-resistance ovarian cancer cells are regulated by the lysosomal function. *Journal of Extracellular Biology*, *3*(6), e157. https://doi.org/10.1002/jex2.157.
  - ⚬ A study showing cisplatin-resistance in ovarian cancer cells leads to an increase in chemo-small extracellular vesicles, which is regulated by a dysfunctional lysosomal system that promotes their secretion over degradation.
- Feng, J., Wang, Z.-X., Bin, J.-L., Chen, Y.-X., Ma, J., Deng, J.-H., Huang, X.-W., Zhou, J., & Lu, G.-D. (2024). Pharmacological approaches for targeting lysosomes to induce ferroptotic cell death in cancer. *Cancer Letters, 587*, 216728. https://doi.org/10.1016/j.canlet.2024.216728.
  - ⚬ A strategy to induce cell death via lysosomal targeting.
- Gagliardi, S., Mitruccio, M., Di Corato, R., Romano, R., Aloisi, A., Rinaldi, R., Alifano, P., Guerra, F., & Bucci, C. (2024a). Defects of mitochondria-lysosomes communication induce secretion of mitochondria-derived vesicles and drive chemoresistance in ovarian cancer cells. *Cell Communication and Signaling : CCS, 22*(1), 165. https://doi.org/10.1186/s12964-024–01507-y.
  - ⚬ Defects in the communication between mitochondria and lysosomes in ovarian cancer cells can lead to the secretion of mitochondria-derived vesicles, which then promote chemoresistance.
- Liu, S., Perez, P., Sun, X., Chen, K., Fatirkhorani, R., Mammadova, J., & Wang, Z. (2024). MLKL polymerization-induced lysosomal membrane permeabilization promotes necroptosis. *Cell Death and Differentiation, 31*(1), 40–52. https://doi.org/10.1038/s41418-023–01237-7.
  - ⚬ A study showing involvement of the lysosome in necroptosis.
- Liu, S., Yao, S., Yang, H., Liu, S., & Wang, Y. (2023). Autophagy: Regulator of cell death. *Cell Death & Disease, 14*(10), 648. https://doi.org/10.1038/s41419-023–06154-8.
- Wu, G. S., Saftig, P., Peters, C., & El-Deiry, W. S. (2025). Correction: Potential role for Cathepsin D in p53-dependent tumor suppression and chemosensitivity. *Oncogene, 44*(9), 630–631. https://doi.org/10.1038/s41388-025–03291-6.
  - ⚬ A study showing a lysosome enzyme, Cathepsin D, in chemosensitivity.
- Xu, J., Gu, J., Pei, W., Zhang, Y., Wang, L., & Gao, J. (2024). The role of lysosomal membrane proteins in autophagy and related diseases. *The FEBS Journal, 291*(17), 3762–3785. https://doi.org/10.1111/febs.16820.
  - ⚬ This study focuses on the lysosome's role in autophagy and its association with various disorders.
- M. A., & Keulers, T. G. (2025). Key Mechanisms in Lysosome Stability, Degradation and Repair. *Molecular and Cellular Biology, 45*(5), 212–224. https://doi.org/10.1080/10985549.2025.2494762.
  - ⚬ This study highlights factors affecting the stabilization and damage of the lysosome that potentially will allow therapeutic intervention.

## Data Availability

Not Applicable.
